# Wavelength-Conversion-Material-Mediated Semiconductor Wafer Bonding for Smart Optoelectronic Interconnects

**DOI:** 10.3390/nano9121742

**Published:** 2019-12-06

**Authors:** Kodai Kishibe, Soichiro Hirata, Ryoichi Inoue, Tatsushi Yamashita, Katsuaki Tanabe

**Affiliations:** Department of Chemical Engineering, Kyoto University, Kyoto 615-8510, Japan

**Keywords:** semiconductor, interface, wafer bonding, frequency conversion, optoelectronics, photonic device, solar cell, photonic integrated circuit

## Abstract

A new concept of semiconductor wafer bonding, mediated by optical wavelength conversion materials, is proposed and demonstrated. The fabrication scheme provides simultaneous bond formation and interfacial function generation, leading to efficient device production. Wavelength-converting functionalized semiconductor interfacial engineering is realized by utilizing an adhesive viscous organic matrix with embedded fluorescent particles. The bonding is carried out in ambient air at room temperature and therefore provides a cost advantage with regard to device manufacturing. Distinct wavelength conversion, from ultraviolet into visible, and high mechanical stabilities and electrical conductivities in the bonded interfaces are verified, demonstrating their versatility for practical applications. This bonding and interfacial scheme can improve the performance and structural flexibility of optoelectronic devices, such as solar cells, by allowing the spectral light incidence suitable for each photovoltaic material, and photonic integrated circuits, by delivering the respective preferred frequencies to the optical amplifier, modulator, waveguide, and detector materials.

## 1. Introduction

**Figure 1:**
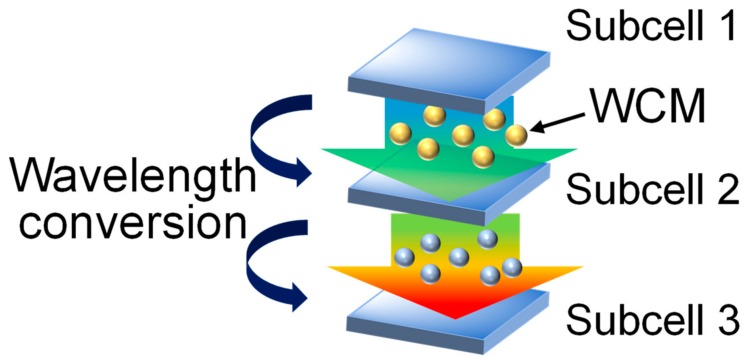
Conceptual drawing of the application of the wavelength-converting material (WCM)-mediated bonding for multijunction solar cells.

The wafer bonding technique [1,2,3] is used to generate semiconductor heterostructures with low defect densities, which are difficult to obtain by the conventional epitaxial growth methods, owing to crystalline lattice mismatches. Therefore, semiconductor wafer bonding is promising for the realization of high-performance optoelectronic components and has been employed to fabricate various heterostructured devices, such as lasers [4,5,6,7], light-emitting diodes [8], photodetectors [9], and solar cells [6,10,11]. Here, we present optical wavelength-converting material (WCM)-mediated wafer bonding, as a means to simultaneously provide bond formation and interfacial function generation. This study is part of a series of developments regarding functional bonding interfaces; we have previously developed graphene-mediated semiconductor wafer bonding [12]. There exist various semiconductor wafer bonding schemes, such as semiconductor-to-semiconductor direct bonding [6,10,11] and oxide- [4,5], metal- [8], and polymer-mediated bonding [7]. However, there has been no WCM-mediated bonding reported so far, to the best of our knowledge. Our novel semiconductor-bonding scheme may improve, for example, multijunction photovoltaic cell efficiencies, by converting the wavelength of the light transmitted through the upper subcell to one that is highly absorbed by the lower subcell [13,14], as conceptually depicted in Figure 1. It also improves the performance of hybrid optical transceivers in photonic integrated circuits [15,16,17,18] by delivering suitable frequencies to each optical amplifier, modulator, waveguide, and detector material. Furthermore, our bonding is performed at room temperature and does not require heating, unlike the conventional wafer direct bonding processes, and therefore exhibits no risk of damaging the active material or retarding the production line.

## 2. Experimental Methods

The polished surfaces of boron-doped *p*-type <100> Si wafers, with a doping concentration of ~1 × 10^19^ cm^−3^ and a thickness of 280 μm, were first coated using photoresist for the purpose of protecting the Si surfaces to be bonded in the dicing process into 64 mm^2^. The diced wafers were dipped in acetone for 5 min to remove the photoresist coating and to degrease the Si surfaces to be bonded. They were then submerged in 9% hydrofluoric acid for 1 min to remove the SiO_2_ native oxide layer formed on the Si wafers. We used a commercially available WCM of 4,7-bis(4-*tert*-butylphenyl)-2-octylbenzotriazole (RAYCREA, Nitto Denko Corp., Osaka, Japan) [19]—a fluorescent dye compound. However, the bare WCM was composed of solid-state particles and therefore could hardly be incorporated stably, in its original form, in semiconductor wafer bonding interfaces. Therefore, we used a hydrogel material as an adhesive and a viscous organic matrix to embed the fluorescent particles. A 2.5 w/v% polyacrylamide (PAM) aqueous solution (aq.) was prepared by mixing PAM powder with deionized water and stirring well to prevent the aggregation of the adhesive PAM particles. WCM, which was ground with a mortar to obtain uniform particle diameters, was mixed with the 2.5 w/v% PAM aq., and five types of mixtures (0, 1 × 10^−4^, 1 × 10^−3^, 5 × 10^−3^, and 1 × 10^−2^ g/mL) were prepared. The prepared hydrogel containing WCM was then uniformly spin-coated onto the Si wafer. For a higher reproducibility of the experimental results, this spin-coating process was repeated three times. The Si piece coated with the hydrogel containing WCM was bonded to a bare Si piece at room temperature under a uniaxial pressure of 0.1 MPaG. Incidentally, the use of a WCM that can be molecularly dispersed in a polymer binder would have been technically more desirable, but such a WCM was not commercially available, and we were not equipped with the ability to synthesize it. Therefore, we employed the particulate WCM in this work.

The normal detachment stresses were measured as bonded interfacial strengths for the bonded samples. We connected the outer surface of the bonded sample to a digital spring weight scaler via a solid wire firmly attached to the sample surface using a household adhesive glue. Then, we pulled the scaler outward in the direction normal to the sample die until the bonded sample was debonded while the weight scaler recorded the maximum force at the point of delamination. For electrical measurements, an Au–Ge–Ni alloy (80:10:10 wt %) and pure Au layers with thicknesses of 30 and 150 nm, respectively, were sequentially deposited on the outer sides of the bonded samples as ohmic electrodes via thermal evaporation. For the interfacial observation, we used an infrared transmission analyzer (IRise-T, Moritex Corp., Asaka, Japan) with an optical wavelength of 1.2 μm. For the optical measurements of WCM, a sample containing 0.03 g/cm^2^ of WCM between glass plates was prepared in the same manner as above but with no surface pretreatment. In this study, for the sake of simplicity, we used Si wafers as the representative semiconductor material, but our concept can be easily extended to other semiconductors, as numerous wafer bonding experimental demonstrations have been reported, thus far, between dissimilar semiconductor materials [4,5,6,7,8,9,10,11].

## 3. Results and Discussion

**Figure 2:**
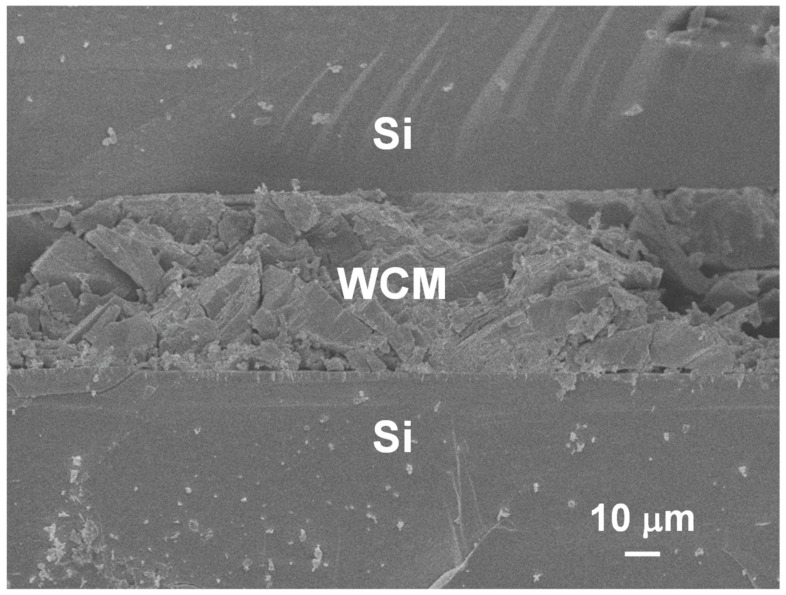
Cross-sectional scanning electron microscope image of a bonded interface (WCM concentration: 1 × 10^−2^ g/mL).

**Figure 3:**
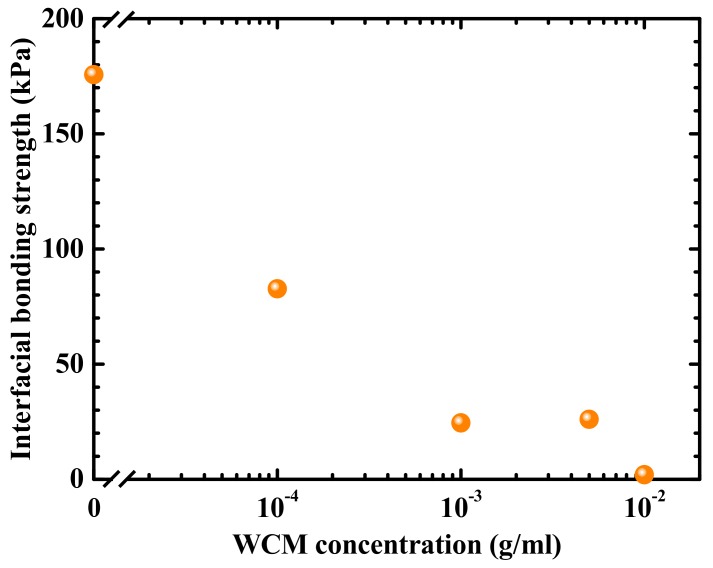
Interfacial bonding strength vs. WCM concentration.

**Figure 4:**
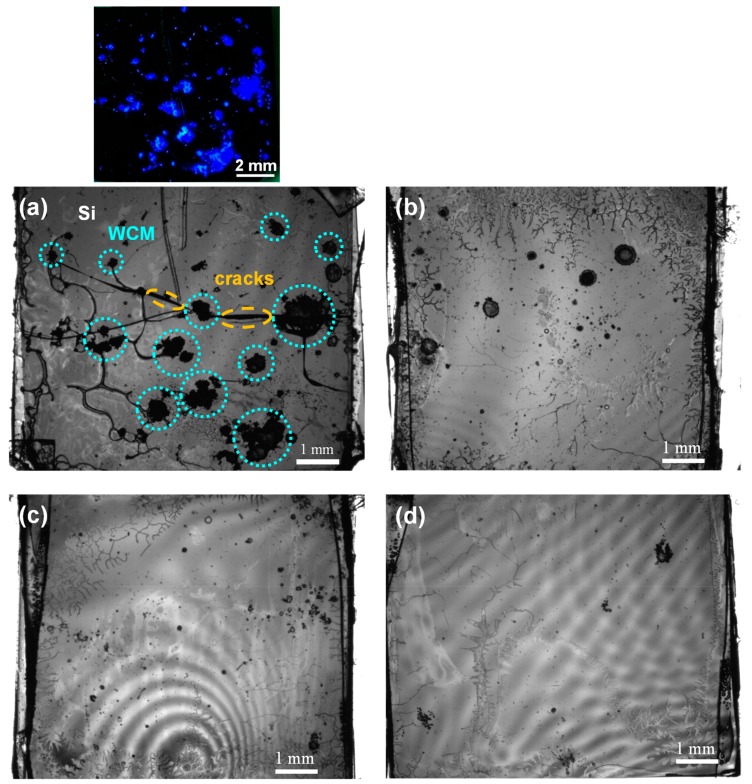
Infrared inner images of bonded interfaces (WCM concentration: (**a**) 1 × 10^−2^ g/mL, (**b**) 5 × 10^−3^ g/mL, (**c**) 1 × 10^−3^ g/mL, (**d**) 1 × 10^−4^ g/mL). The accompanying image (top) is an optical photograph of the debonded Si surface after the intentional debonding of the bonded sample of (**a**) under an ultraviolet lamp.

Figure 2 shows a cross-sectional scanning electron microscope image of the bonded interface (WCM concentration: 1 × 10^−2^ g/mL). As observed in this image, the wafers are firmly and uniformly in contact with each other, with sufficient mechanical strength to endure the cleavage of the bonded sample. The WCM-containing interlayer in this example may seem relatively thick, considering the field of thin-film optoelectronic devices. According to the required degree of the wavelength-converting function and interfacial mechanical stability, the interlayer thickness can be controlled in each application. As a general design direction, a thicker interlayer provides a sufficient wavelength conversion efficiency and higher bondability by mitigating the semiconductor surface roughness, whereas a thinner interlayer supports the advantages in the device weight, production cost, and throughput. The WCM uniformity in the interlayer may be improved by increasing the dispersion of the WCM particles, as discussed later. We measured the dependence of the bonding strength on the WCM concentration; the results are shown in Figure 3. At the present stage, the experimental reproducibility of the bonding strength is relatively low, and we therefore plotted the best measured values for each bonding condition. As observed in these results, the majority of the samples exhibited sufficient bonded interfacial strength to endure a series of optoelectronic device fabrication processes and user operations. The hydrogen bonds stemming from PAM presumably cause the adhesion to the semiconductor surfaces [20,21]. More specifically, hydrogen bonds may form between the –NH_2_ groups of PAM and the Si surface terminated by –OH groups, owing to the water contained in PAM [20]. In addition, the PAM matrix holds the WCM particles, suppressing their sedimentation. This is due to its viscosity, induced by the entanglement of PAM polymer chains. The results shown in Figure 3 indicate that, as the concentration of WCM increases, the interfacial bonding strength decreases. To further analyze the cause of this result, we observed the inner structure of the sample (WCM concentration: 1 × 10^−2^ g/mL) using an infrared transmission analyzer (Figure 4a). The black spots in Figure 4a identify the WCM via the accompanying emission image, and this image verifies that the WCM particles are not dispersed uniformly; rather, they appear aggregated. This random dispersion of WCM is due to our having no technical control of the spatial distribution of WCM at the present stage and also, presumably, to the energetic stability in the aggregated state of the WCM particles. Such clustered WCM particles may cause intense roughness that the PAM cannot mitigate. Therefore, the interfacial bonding strength significantly decreases at higher concentrations of WCM, as we observed larger aggregates for higher WCM concentrations (Figure 4). In addition, the wafers may experience serious damage, as evidenced by the cracks observed in Figure 4. This applies especially to higher WCM concentrations, because the bonding pressure is concentrated in particular areas of the agglomerated particles. Therefore, to improve the properties of our WCM-containing interfaces—such as the mechanical strength, electrical conductivity, and optical transparency—it may be effective to increase the dispersion of WCM by selecting the optimum preparation process conditions, such as the organic solvent species, the spin-coating rotation velocity, and the coating repetition. To account for the WCM concentration vs. the interfacial stability trade-off observed in Figure 3, it is important to adopt the optimal organic species and thickness of the matrix and the dispersion status of the WCM particles, according to the property requirements for each application. For instance, a design principle can be described as follows: a relatively thick interlayer with low-concentration WCM can fulfill the demands of both high mechanical strength and high optical conversion efficiency.

**Figure 5:**
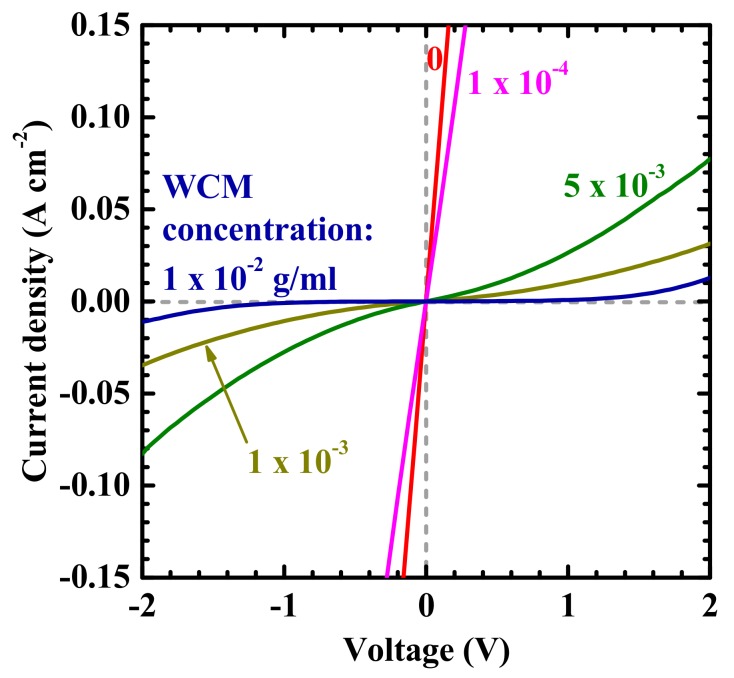
Interfacial current–voltage (*I*–*V*) characteristics on WCM concentration.

Figure 5 shows the current–voltage (*I*–*V*) characteristics of the bonded interfaces for various concentrations of WCM. An ohmic interfacial electrical conductance with relatively low resistivity is observed when bonding with lower WCM concentrations. PAM, which is a hydrophilic polymer material, can contain water internally, and the water-originated ions are known to act as electrical carriers and provide electric conductance [21]. Intuitively, one might think that the presence of water has a negative effect on electronic component fabrication. However, in hydrogels, the water molecules are stably embedded in the matrices of organic polymer chains, and hydrogel-based devices have been successfully fabricated and operated [21], as in the field of organic electronics. As the concentration of WCM increases, the conductivity decreases, presumably because the highly agglomerated WCM for high WCM concentration conditions can induce voids between the wafers, as indicated in Figure 4. However, the strong decrease of the electrical conductivity with the increased concentration of WCM cannot be explained simply by a decreased width of the conductive joint, because the observed decrease is much larger than the loss of the joint area represented by Figure 4. The bonding interlayer thicknesses for the samples with WCM concentrations of 0 and 1 × 10^−2^ g/mL were measured at about 5 and 50 μm, respectively, by scanning electron microscopy. The interlayer thickness increase with WCM concentration can be attributed to the generation of larger WCM aggregates for higher WCM concentrations, as observed in Figure 4. Consequently, such large, and therefore tall, aggregated WCM clusters may mechanically resist the interlayer compression through the applied pressure in the bonding process. In this way, the bonding interlayer thickness becomes significantly larger for higher WCM concentrations. Overall, the unintentional combinative effect of such a decrease in the area and an increase in the length of the electrically conductive channel may have resulted, in our experiments, in the significant decrease of the interfacial electrical conductivity with WCM concentration. An increase in dispersion may be effective in obtaining, simultaneously, a high WCM concentration and a high electrical conductivity. A similar conclusion was reached in the previous case of WCM concentration vs. mechanical stability investigation, although the thickened dilute WCM interlayer strategy may also be applicable in this case. In addition, WCM particles with sizes much smaller than the total interlayer thickness may improve the density–conductivity trade-off. Moreover, the doping of electroconductive polymers, such as polyacetylene or polythiophene [22], in PAM may work effectively to produce a higher electrical conductivity.

**Figure 6:**
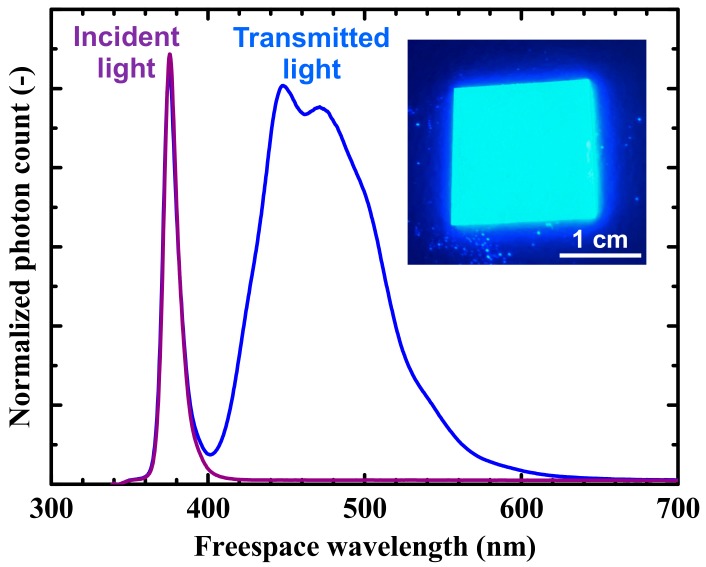
Transmission spectrum of the bonded samples with WCM and spectrum of the incident light. Inset: optical photograph of the bonded sample with WCM under an ultraviolet lamp.

The inset of Figure 6 shows an optical photograph of the bonded glass plate sample, prepared for optical transmission measurements, under an ultraviolet lamp. In the snapshot of the inset of Figure 6, it is distinctly observed that the WCM at the bonded interface between the glass plates converts the incident ultraviolet light into visible blue light output. The transmittance spectrum of the bonded glass samples with WCM, along with the spectrum of the incident light, is shown in Figure 6. Note that, in our fiber-coupled optical transmission measurement setup, the sample-transmitted light scattered by the WCM particles is poorly coupled to the detection spectrometer, and therefore, the integration times of the spectrometer were adjusted so as to enable a reasonable comparison between the incident and the transmitted light. The change in the spectrum of the photon count in Figure 6 verifies that the WCM embedded in the bonded interface converted the incident light peaking at approximately 380 nm into another bundle of light peaking at approximately 460 nm. Such an optical wavelength conversion can be utilized, for instance, in solar cell applications, where the interfacial WCM absorbs an ultraviolet light (380 nm) and emits a visible light (460 nm) to fit the solar spectral irradiance to the spectral sensitivity of crystalline silicon absorption. This particular WCM can thus be utilized, for example, at the interfaces of (Al)(In)GaN/Si-based multijunction solar cells for the photocurrent improvement of the Si subcells. However, our WCM-bonding scheme can be used regardless of the material species, and thus, the most suitable combination of semiconductors can be selected, on demand, for each application situation. For example, stacked hybrid optical transmitters [4,7] in photonic integrated circuits could benefit from down-converting WCMs for longer wavelengths by enabling the use of GaAs-based or InP-based high-power diode lasers emitting at the red to the 900 nm region to be converted into the optimal wavelengths for the Si-based optical waveguides or silica-based optical fibers (1.3–1.6 μm).

## 4. Conclusions

In this study, we proposed and experimentally validated WCM-mediated semiconductor wafer bonding. This scheme could simultaneously support bond formation and interfacial function generation and thus potentially realize low-cost and high-throughput device production. The wavelength-converting heterointerfaces could improve, for example, multijunction solar cell efficiencies via photon management and current matching among the subcells and hybrid photonic-integrated circuitry performances by delivering the most suitable frequencies to each optical transceiver component. The bonding was carried out in ambient air at room temperature and therefore provides a cost advantage with regard to device manufacturing. We used Si wafers as the representative semiconductor material, for the sake of simplicity, but our concept can be extended to other semiconductors, as numerous wafer bonding experimental demonstrations between dissimilar semiconductor materials have been reported thus far [4,5,6,7,8,9,10,11]. We demonstrated the use of a down-conversion material in this study, but our bonding scheme can also be applied to up-conversion materials based, for instance, on harmonic generation [23,24] or triplet–triplet annihilation [25,26], for the efficient up-conversion of optical irradiance with a relatively low intensity, such as sunlight.
